# Editorial: Management and prevention of long-term complications related to the niche in the uterine cesarean section scar

**DOI:** 10.3389/fsurg.2024.1381449

**Published:** 2024-02-21

**Authors:** Michał Pomorski, Piotr Sieroszewski

**Affiliations:** ^1^ II Department of Gynecology and Obstetrics, Wroclaw Medical University, Wrocław, Poland; ^2^ Department of Fetal Medicine and Gynecology, Medical University of Lodz, Łódź, Poland

**Keywords:** cesarean section, long-term complications, niche, istchmocele, niche surgery

**Editorial on the Research Topic** Management and prevention of long-term complications related to the niche in the uterine cesarean section scar

Cesarean section [CS] is the most common major surgical procedure performed worldwide. According to WHO currently 20% of deliveries are by cesarean section, while in the European Union this percentage has already reached 30% (1).

Like any surgery, CS has its bright and dark sides. The positive aspect of CS is that it allows safe delivery in the event of maternal and/or fetal complications and contraindications to vaginal delivery.

Cesarean section should be seen as an intervention that saves life and health of mother and fetus.

However on the other hand CS is associated with short- and long-term complications.

Short-term complications, as with any abdominal surgery, include postoperative pain, complications of anesthesia, surgical wound infection, thromboembolism, haemorrhage and many others.

Long-term complications are much more specific to CS. They include complications related to the uterine CS scar, and abdominal CS scar.

Uterine scar complications after CS are related to incomplete healing of the uterine wall at the incision site. This incomplete healing leads to formation of a niche. A niche is defined by the European Niche Taskforce as an indentation at the site of a Cesarean section scar that is at least 2 mm deep (2).

The presence of an incompletely healed uterine CS scar (i.e., a scar with a niche) can cause complications both in subsequent pregnancies and in non-pregnant women.

In pregnant women, these complications may be life-threatening and include cesarean scar pregnancy, cesarean scar dehiscence or rupture and placenta previa accreta (3, 4). In non-pregnant women the presence of a niche leads to a group of symptoms recently named Cesarean Scar Disorder (CSDi) (5). CSDi includes such symptoms as: postmenstrual spotting, pain during uterine bleeding, technical issues with catheter insertion during embryo transfer and unexplained subfertility. Given the increasing number of CS, and thus the growing population of women with incompletely healed CS scars, measures should be taken to adequately diagnose and treat the uterine niche.

Currently, the standard diagnosis of niche includes transvaginal ultrasonography, including 2D, 3D techniques and sonohysterographic approaches on a non-pregnant uterus (6).

Methods of treatment of symptomatic niche have recently been developed and include laparoscopic/robotic or transvaginal niche repair and hysteroscopic niche resection (7).

An article by Xia et al. published under this research topic compares the effectiveness of transvaginal niche repair with hysteroscopic resection [Comparative effectiveness of transvaginal repair vs. hysteroscopic resection in patients with symptomatic uterine niche]. This study showed that both procedures lead to reduction in CSDi symptoms and a reduction in the niche as an anatomic structure. The advantage of transvaginal niche repair is the thickening of the myometrium at the scar site, which could potentially have a positive effect on the scar strength during subsequent pregnancies. However, hysteroscopic procedure is associated with fewer complications, shorter operating time and shorter hospital stay.

A rare but serious complication associated with uterine CS scar is a cervicovesical fistula. The fistula is most often the result of an intraoperatively unrecognized bladder injury. The most common risk factor are adhesions from previous CS procedures. Symptoms can vary depending on the location and size of the fistula and mainly include hematuria during menstrual bleeding and transvaginal urine leakage. A case report by Sun et al. describes a cervicovesical fistula with unusual symptoms [A neglected cervicovesical fistula diagnosed and repaired by combined hysteroscopy and laparoscopy: A case report and review of literature]. A comprehensive description of the fistula repair procedure is also provided, along with a review of literature.

Another group of long-term CS complications are those associated with abdominal CS scar.

A serious complication in this group is scar endometriosis, also referred to as abdominal wall endometriosis (AWE). AWE results from direct implantation of endometrial tissue into abdominal wall structures at the time of CS. The AWE develops in only 0.03%–1% of women after CS (8). However, given that 30 million CS are performed annually worldwide, the population of women with AWE is growing rapidly. Symptoms mainly include a palpable tumor and cyclic pain. Most AWE is localized in the subcutaneous fat tissue above the fascia, but there is a growing number of patients with extensive disease in which AWE involves the fascia, abdominal muscles, peritoneum and even bladder wall. The surgical management of advanced AWE, along with a review of the literature, is presented in this series of articles in a manuscript submitted by Triantafyllidou et al. [Surgical management of abdominal wall sheath and rectus abdominis muscle endometriosis: a case report and literature review].

Another complication associated with abdominal CS scar is the very rare possibility of IUD migration from the perforated uterine cavity into the abdominal wall structures. The mechanism of migration, as well as management, is discussed in the study by Jing [Case report: An intrauterine device hugging the musculus rectus abdominis through the center of a cesarean scar].

The last 2 articles published under this research topic describe the management of early complications associated with CS. In a randomized controlled trial Yang et al. investigated the effect of glucose-insulin-potassium (GIK) therapy on uterine cramping pain (UCP) after CS [Glucose-insulin-potassium alleviates uterine cramping pain following cesarean delivery: A randomized, controlled trial]. The main cause of pain after CS is related to the wound created in the abdominal wall worsening with uterine contractions. Recently, significant progress has been made in postoperative analgesia related to the abdominal wall wound through the widespread introduction of transversus abdominis plane (TAP) block (9). This procedure involves injecting a local anesthetic under ultrasound guidance into the space between the transversus abdominis muscle and the oblique abdominal muscle. However, TAP block has no effect on the pain associated with uterine contractions. The GIK therapy proposed by Yang et al. may be a promising complementary analgesic therapy for treatment of pain associated with uterine cramping.

The final article in this series by Song et al. presents a case of prolonged neuromuscular block after emergency CS performed under general anesthesia in a woman treated with magnesium sulfate and calcium channel blocker for severe preeclampsia [Postoperative residual neuromuscular block in a woman with severe preeclampsia treated with magnesium sulfate and nicardipine: A case report and literature review]. Since preeclampsia affects 3%–8% of pregnant women, both obstetricians and anesthesiologists should be aware of this possible complication.
